# Using technology to improve access to optometric services

**Published:** 2022-06-07

**Authors:** Jessica Massie, Priya Morjaria

**Affiliations:** 1 Freelance Global Eye Health Consultant and Public Health Optometrist, Australia.; 2 Assistant Professor and Public Health Optometrist: London School of Hygiene & Tropical Medicine and Head of Global Programme Design: Peek Vision, UK.

**Teleoptometry can reduce barriers and improve access to primary eye care services delivered by optometrists.**

**Figure:**
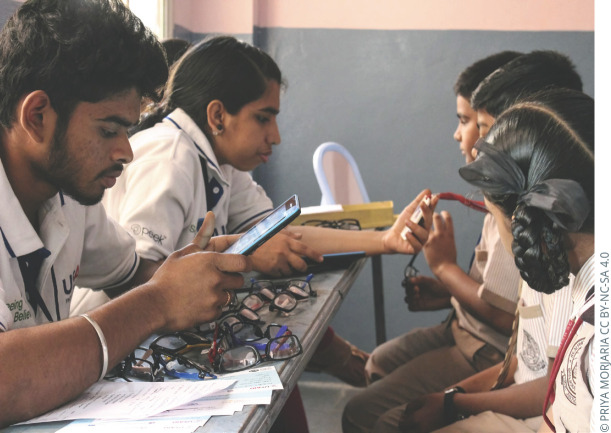


Measures to reduce the transmission of COVID-19 have severely limited the number of people who could make use of in-person optometry services over the last two years, leading many service providers to try and bridge the gap by using different forms of communication technology to improve access. For example:

- the deployment of technicians to facilitate remote optometry examinations in the community, e.g. by assessing vision and capturing patient details using mobile apps
- offering telephone or video consultations as a form of triage to streamline access to timely care for patients with urgent presentations
- offering such ‘remote’ or ‘teleoptometry’ consultations to existing patients who need follow-up care and advice, e.g., patients with low vision.

Teleoptometry can be a valuable companion to in-person services. Although peer-reviewed literature about this is limited, we know that in settings where optometrists are involved in online referrals that include video consultations, patients report being highly satisfied and accepting of these services.^1^ Similarly, patients receiving low vision services via teleoptometry have reported a high level of satisfaction with the consultation.^2^

Some of the challenges that have arisen include uncertainty regarding professional indemnity insurance, and ensuring that teleoptometry services are delivered in accordance with local privacy and information security laws. The College of Optometrists in the United Kingdom have published temporary guidelines to guide optometrists conducting remote consultations during the COVID-19 pandemic; this is accompanied by a clinical telephone review template.^3^

## What remains a challenge?

More broadly, concerns have been raised about the delivery of teleophthalmology services increasing inequity in marginalised, vulnerable populations due to the ‘digital divide’ – the gap between those who have ready access to the internet and electronic devices, and those who do not.^4^ It is therefore essential to design teleoptometry services that prevent the further marginalisation of vulnerable groups, for example by ensuring access to language interpretation or sign language services.

Teleoptometry services created during the pandemic present a unique opportunity to continue offering access to primary eye care services delivered by optometrists, which will help to reduce the barriers to access faced by marginalised and vulnerable populations. However, there is an urgent need for more evidence to support the safe, effective and equitable delivery of teleoptometry practice, including potential solutions to the digital divide.

**How to carry out a remote telephone/video optometry consultation**

During the COVID-19 pandemic, the College of Optometrists, UK, published the following steps for optometrists to follow when carrying out remote telephone or video consultations. Adapted from: *Remote consultations during COVID-19 pandemic* (https://bit.ly/RemoteOptom).

1. Verify the patient's identity and contact details.
2. Ensure you and the patient are in a private space, as you would in an in-person consultation.
3. Confirm the patient is happy to continue with the virtual review.
4. Utilise the clinical telephone review template to document the conversation, including any observations you make during the video call.
5. Determine the management category for the patient: 1) refer to eye casualty for sight/life threatening condition, 2) book urgent optometry review, or refer to eye casualty for potentially sight/life threatening eye condition, 3) advise to self-manage minor eye condition, 4) book appointment for non-urgent eye condition.
6. Support self-care by emailing or posting advice to patients.
7. Securely store the clinical record of the telephone or video consultation.
8. Advise the patient to contact you again should their symptoms worsen.
